# One health: the importance of companion animal vector-borne diseases

**DOI:** 10.1186/1756-3305-4-49

**Published:** 2011-04-13

**Authors:** Michael J Day

**Affiliations:** 1 School of Veterinary Sciences, University of Bristol, Langford BS40 5DU, UK

## Abstract

The international prominence accorded the 'One Health' concept of co-ordinated activity of those involved in human and animal health is a modern incarnation of a long tradition of comparative medicine, with roots in the ancient civilizations and a golden era during the 19^th ^century explosion of knowledge in the field of infectious disease research. Modern One Health tends to focus on zoonotic pathogens emerging from wildlife and production animal species, but one of the most significant One Health challenges is rabies for which there is a canine reservoir. This review considers the role of small companion animals in One Health and specifically addresses the major vector-borne infectious diseases that are shared by man, dogs and cats. The most significant of these are leishmaniosis, borreliosis, bartonellosis, ehrlichiosis, rickettsiosis and anaplasmosis. The challenges that lie ahead in this field of One Health are discussed, together with the role of the newly formed World Small Animal Veterinary Association One Health Committee.

## One Health: History and Scope

The 'One Health' concept states simply that there should be a seamless interaction between veterinary and human medicine with clinicians, researchers, agencies and governments working together for the benefit of domestic and wild animal and human health and the global environment. Such interactions may take place at many levels - from management of zoonotic infectious disease outbreaks in the field, to joint research programmes to integrated policy making and funding decisions.

The concept of One Health is nothing new. Dunlop and Williams in their *History of Veterinary Medicine *[1] catalogue the early recognition of the significance of zoonotic infectious disease. The ancient Mesopotamians collated laws into codes and the Eshuna Code of 2300 BC makes the owner of a rabid dog responsible for the containment of that animal. The Babylonian king Adad-apla-iddina (1068-1047 BC) constructed a temple to the healing goddess Gula who was represented with or as a dog and was worshiped as a healer and protector from rabies. It is also believed that the role of the dog as an adjunct to healing was recognized through the ability of these animals to provide comfort to patients. The ancient Egyptians provide the first documentation of animal diseases in the Kahun papyrus (1900 BC) and in the context of the present review; it is believed that this civilization had awareness of vector-borne disease through their use of mosquito netting. There is a rich history of comparative medicine within the ancient Chinese dynasties in which there were organized specialities of physicians, surgeons, dieticians and veterinarians - clearly emphasizing the importance of animals to this society. The principles of yin-yang as practiced through acupuncture applied equally to human and animal patients. In ancient Greece, Aristotle (384-322 BC) continued the tradition of Hippocrates (460-367 BC) and promoted comparative medicine through his extensive studies of animal anatomy and the pathology of animal diseases. This tradition of comparative anatomy was continued in ancient Roman civilization with the most prominent contribution made by Galen (130-200 AD). The Hippocratic-Galenic principles continued throughout Medieval Europe to the Renaissance where the comparative anatomical legacy of Leonardo da Vinci (1452-1519) laid the way for future developments. Later, John Hunter (1728-1793) became a true practitioner of comparative medicine and was tutor to Edward Jenner (1749-1823) whose development of vaccination was a classical example of the One Health principle. The formalization of One Health is often attributed to Claude Bourgelat, the founder of the first veterinary school in Lyon in 1761, precisely 250 years ago. In writing on the relationship between human and animal medicine, Bourgelat stated that 'either medicine will mutually enlighten and perfect the other when we discard a derisory, harmful prejudice'. The greats of the golden era of infectious disease research - Louis Pasteur (1822-1895), Robert Koch (1843-1910), Rudolf Virchow (1821-1902) and John M'Fadyean (1853-1941), were all proponents of One Health and many of their advances were based in studies of animal disease [1-3].

It was perhaps in the 20^th ^century that the medical and veterinary professions became slightly distant, a fact that was recognized by Calvin Schwabe (1927-2006) who promoted a 're-unification' from the 1960s and is attributed with coining the term 'One Medicine' or One Health [3,4]. It was however, not until the past five years, that the One Health concept has truly gathered international momentum. Collaborative ventures between the British Medical Association and the British Veterinary Association [5] and the American Medical Association and the American Veterinary Medical Association [6] paved the way for the seminal concept note produced following a meeting of the World Organization for Animal Health (OIE), the World Health Organization (WHO), the Food and Agriculture Organization (FAO), the United Nations Children's Fund (UNICEF) and the World Bank http://www.oie.int/fileadmin/Home/eng/Current_Scientific_Issues/docs/pdf/FINAL_CONCEPT_NOTE_Hanoi.pdf. A number of organizations have endorsed the One Health concept (e.g. the Federation of Veterinarians of Europe) and One Health groups have been established; including the One Health Commission http://www.onehealthcommission.org/, the One Health Initiative http://www.onehealthinitiative.com/index.php and the Comparative Clinical Science Foundation http://www.onemedicine.org.uk/. These initiatives have culminated recently in the First International One Health Conference, held in Melbourne, Australia.

## Companion animals in One Health

From the beginnings summarized above, One Health has now expanded rapidly with endorsement by numerous medical and veterinary organizations. The major focus of these proponents has been the very high-impact interactions between human and production animal and wildlife health with global zoonotic disease pandemics and 'emerging' infectious diseases deemed to have arisen in these animal species. Many of the best examples of such infections are viral, where virus mutation or re-assortment permits extension of the target host range (e.g. SARS coronavirus, H5N1 and H1N1 influenza virus, Nipah virus, Hendra virus, human immunodeficiency virus [HIV]), but the role of parasitic diseases in One Health has also been discussed [7,8]. The impact of environmental change (e.g. climate change, deforestation and urbanization) and lifestyle change including the increase in global human and animal movement [9] has also become part of the One Health concept.

Perhaps less often considered has been the enormous potential role of companion animals, and particularly the domesticated dog and cat, in One Health. In developed nations, pet ownership has reached unprecedented levels and these animals play a significant role in family life. In 2006, there were 72 million pet dogs in the USA (in 37% of households) and 81 million pet cats (in 32% of households) (2007 US Pet Ownership and Demographics Sourcebook). Estimates for 2010 in the UK are for 8 million pet dogs (in 23% of households) and 8 million pet cats (in 19% of households) http://www.pfma.org.uk. Increasingly over past decades, the small companion animal spends a majority of its life within the indoor domestic environment in very close physical association with its owners. Although many of these animals will enjoy a very high standard of healthcare with increasing longevity, there are a number of zoonotic infectious diseases that may be transmitted directly or indirectly from these species. Transmission of such diseases is dependent upon the lifestyle of the pet and is influenced by factors such as vaccination and parasite control, exposure to other domestic animals or wildlife (including urban wildlife such as foxes or small rodents) or exposure to particular environments (e.g. exercise in wooded areas with questing tick populations). The keeping of domestic companion or working small animals is not solely linked to affluence - in most developing nations there is individual or communal village ownership of dogs and cats, and in most of these countries there are extraordinary numbers of feral dogs and cats that have intensive contact with the human urban environment. In contrast, the majority of these animals may never receive veterinary attention and remain significant reservoirs of zoonotic infection.

There is no better example of this than rabies infection, which despite its long historical documentation, remains as much an issue today as at any time in history. Recent WHO estimates suggest that at least 55,000 people die each year from rabies infection; that 95% of these deaths occur in the developing nations of Asia and Africa and that 99% of cases are transmitted by dogs [10]. The significance of rabies virus infection is promoted through initiatives such as World Rabies Day http://www.worldrabiesday.org/ and the Afya vaccination programme in Africa http://www.afya.org/ and in September 2011 the OIE, WHO and FAO will host a Global Conference on Rabies Control in Seoul, Republic of Korea.

Less well-publicized are the remaining infectious diseases shared by man and dogs and cats that are well-summarized in a new text edited by Rabinowitz and Conti [11]. The present review is written in association with the 6^th ^World Forum on Canine Vector-Borne Diseases and those of zoonotic potential will be highlighted in the section following.

## Companion animal vector-borne diseases in One Health

The major vector-borne infectious diseases of dogs and cats that also infect man are summarized in Table 1. Of single greatest significance is zoonotic visceral leishmaniosis caused by *Leishmania infantum *(*L. chagasi*) for which the domestic dog is the major reservoir for human infection via sandfly transmission. The disease is endemic in many countries throughout southern Europe, the Americas, northern Africa and Asia and high proportions of dogs in these areas are exposed to and often infected by the pathogen. Recent WHO estimates suggest that worldwide up to 12 million people are infected, with 2 million new cases identified annually and a population of some 350 million people at risk of infection; the risk of infection is significantly enhanced in HIV-infected individuals [12]. Control of leishmaniosis represents one of the greatest challenges for One Health and provides a clear example for the necessity of human and veterinary medicine to work together to develop strategies for management and elimination of this disease. Such measures have already occurred in the endemic focus of disease occurring in Brazil where the Ministry of Health oversees a programme of serologically testing dogs and culling positive animals [13]. Two commercially available vaccines for dogs have become available in Brazil and should form an important part of the control strategy. Data have already been published that show that where vaccination is widely practiced, the prevalence of both canine and human infection decreases due to reduced transmission of the organism [14].

**Table 1:** Canine and feline vector-borne diseases that also infect man

Disease	Vector
Leishmaniosis	Sand fly

Borreliosis	Tick

Bartonellosis	Flea Tick

Ehrlichiosis	Tick

Rickettsiosis	Tick Flea

Anaplasmosis	Tick

Dirofilariosis	Mosquito

Yersiniosis	Flea

Tularaemia	Tick

Coxiellosis	Tick

Tick-borne encephalitis	Tick

Louping ill	Tick

West Nile virus encephalitis	Mosquito

Trypanosomiosis	*Triatoma *bugs

A major concern is the extension of traditional endemic areas for *Leishmania *infection, which is now reported as autochthonous disease in northern European countries and North America [15-18]. This is associated directly with the increased mobility of pet animals across borders [16]. Pet travel has been responsible for establishment of reservoirs of Leishmania-infected dogs in these non-endemic areas and has incidentally contributed to the widening geographical range of arthropod vectors (e.g. the establishment of *Rhipicephalus sanguineus *in northern Europe). The unprecedented scale of global companion animal travel is readily demonstrated by the success of the European pet passport scheme. Since its inception in 2000 and up to August 2010, the number of pet animals entering or re-entering the UK alone was 717, 965 http://www.defra.gov.uk/. The focus of attention for health certification for international pet travel has been rabies vaccination (with some countries also requiring evidence of seroconversion after vaccination); however, apart from certain national requirements for tick and tapeworm treatment there has been little consideration of the spread of other zoonotic infectious diseases. Some exceptions to this rule exist; for example importation of dogs into Australia requires testing for monocytic ehrlichiosis, brucellosis, leishmaniosis and leptospirosis, but without such controls there is clear evidence of importation of infection from endemic to non-endemic areas [15]. Global pet travel therefore creates the potential for rapid dissemination of zoonotic infection and represents another major challenge for One Health.

Borreliosis (Lyme disease) is a significant disease of people in endemic areas (particularly in Europe and North America) associated with avian and wildlife reservoirs and *Ixodes *spp. ticks. The major species infecting man and companion animals are *Borrelia burgdorferi *sensu stricto (in North America) and *B. garinii *and *B. afzelii *(in Europe) [19]. Infected dogs and cats pose minimal threat to man, but do provide a means by which infected ticks can be carried into the domestic environment. There is also a risk of human infection should ticks be crushed during removal from a pet animal and tick salivary gland material be exposed to wounds on the hands of an owner. The dog in particular might be employed as a 'sentinel' for monitoring the risk of human disease in an endemic area [20].

Bartonellosis should be regarded as one of the major potential emerging infections of man. Most is known about 'cat scratch disease' caused primarily by *Bartonella henselae *acquired directly from an infected cat and mostly likely transmitted between cats by the cat flea *Ctenocephalides felis *[21-23]. This infection becomes particularly significant in immunocompromised individuals (e.g. HIV-infected individuals with acquired immunodeficiency syndrome [AIDS], those receiving cancer chemotherapy or immunosuppression for transplantation or immune-mediated disease). The scale of the potential problem can be estimated by considering the extraordinary numbers of pet household cats (see above) and their very close physical association with owners, coupled with knowledge that in developing countries the prevalence of seropositivity amongst cats is on average 27% and the presence of active bacteraemia around 10% [24]. The major canine pathogen of these taxa is the tick-transmitted *B. vinsonii *subsp. *berkhoffii*, but a range of other *Bartonella *species are also reported from cats and dogs (and potential arthropod vectors) associated with a spectrum of clinical diseases. Attention is now focussed on these organisms due to a series of investigations showing that they may be associated with a wide range of human syndromes that were often considered chronic idiopathic diseases [21,25]. There remains much to learn about the transmission of *Bartonella *from companion animals to man, but veterinary professionals have an occupational exposure risk due to their frequency of exposure to animal bites, arthropods, arthropod faeces and animal bodily fluids [21]. A target for One Health programmes should be further exploration of the significance of these pathogens in animal and human medicine.

A number of ehrlichial and rickettsial infections are shared by man and companion animals [26]. The cause of canine monocytic ehrlichiosis (*Ehrlichia canis *transmitted by *Rhipicephalus sanguineus*) is not considered an organism with significant zoonotic potential (although human cases of infection are reported); however, human and canine infections with *E. chaffeensis *and *E. ewingii *(transmitted by *Amblyoma americanum*) are well-documented although it is not clear whether the dog acts as a reservoir for human infection. Of increasing significance in human, canine and feline populations is the *Ixodes*-transmitted cause of granulocytic 'ehrlichiosis' (*Anaplasma phagocytophilum*). The reservoir species for this infection are wild rodents and wild and domestic ruminants, but human and companion animal infections are emerging as a significant problem in Europe and North America. The spotted fever group of rickettsiosis are also significant problems for man and dogs. In the Americas, Rocky Mountain spotted fever caused by *Rickettsia rickettsii *(transmitted by *Dermacentor andersoni*, *D. variablis*, *R. sanguineus *and *Amblyomma cajennese*) is the most important of this group, while in Europe, Asia and Africa, Mediterranean spotted fever caused by *R. conorii *(transmitted by *R. sanguineus*) is of greatest concern. The role of the dog in these human infections is again in transporting infected ticks into the domestic environment and the risk to owners of removing infected ticks from their pets. The early diagnosis of canine rickettsiosis by the veterinarian is of importance as this may precede the recognition of the pathogen in in-contact people. The flea-transmitted rickettsioses (*R. typhi *and *R. felis*) also fall into this group [23]. The reservoir potential for domestic pets for *R. typhi *is not proven, but there is much recent interest in the emergence of *R. felis *which is found in dogs, cats and cat fleas (*Ct. felis*) [23,27]. As *R. felis *may be transmitted transovarially and trans-stadially, the domestic pet may introduce infected fleas into the home environment, which then become the means for human infection.

Table 1 also lists a range of other vector-transmitted diseases shared by man and companion animals, a number of which are relatively uncommon causes of human infection, but are important in animal species (e.g. dirofilarasis, West Nile fever).

In summary, the overall challenges for the One Health agenda in addressing these vector-borne diseases are several: (1) promoting awareness and ability to recognize these diseases by the human and veterinary medical professions, (2) undertaking 'joined-up' research programmes that investigate the agents, their vectors and epidemiology, geographical distribution, clinical significance and pathogenesis, (3) developing robust diagnostic tests and surveillance systems for mapping these infectious agents and their vectors globally, (4) identifying diseases for which domestic pet animals are true reservoirs of human infection (e.g. leishmaniosis) and formulating public health strategies that effectively control the disease in the reservoir (e.g. stray dog control, ectoparasiticide treatment, vaccination) in addition to the human patient, (5) investigating the contact between pet animals and peridomestic wildlife species that may act as true reservoirs of these diseases and developing strategies to minimize such contact, (6) promoting awareness of these diseases in the pet-owning public and the importance of regular ectoparasite control programmes for their pets and their domestic environment, and (7) identifying the risks and challenges imposed by increasing global mobility of pet animals and developing strategies to minimize the associated movement of zoonotic infectious disease.

## Companion animal One Health: the WSAVA One Health Committee

Given the growing importance of the One Health concept the World Small Animal Veterinary Association (WSAVA) has recently established a One Health Committee, which has as its remit the firm positioning of small companion animals within the global One Health programme [28]. The committee is chaired by the author and includes academic experts on small animal zoonotic infection (including rabies, leishmaniosis and vector-borne diseases), first opinion veterinary practitioner representation and delegates from the OIE and Centers for Disease Control and Prevention (CDC) in the USA. The committee is funded by the WSAVA Foundation through sponsorship by a consortium of the pet food and small animal pharmaceutical industry and commenced a three year programme of work in January 2011.

Although a major focus of the work of the One Health Committee will be zoonotic infectious disease, including those reviewed in this paper, there are two further key areas in One Health in which small companion animals should play a major role. The first of these is in the field of comparative and translational research. The historical perspective given earlier indicates clearly how throughout history human medicine has benefitted from comparative veterinary studies. To an extent, we seem to have forgotten this legacy and over recent decades research efforts have been polarized and veterinary science been very much the 'poor relation' of its larger brother. It is unquestionable that studies of spontaneously arising disease in relatively outbred and long-lived animals that so closely share our domestic environment must provide information of benefit to human medicine. The availability of the canine and feline genomes [29,30] and the development of microarray genomic screening tools [31,32] provide us with unprecedented ability to explore the basis of canine and feline diseases that so closely mimic those that occur in man. Promoting comparative clinical research will be the second major focus of the WSAVA One Health Committee.

The final aspect of the work of this group will be in supporting the most visible and simple aspect of the interaction between man and companion animals. The ancient Mesopotamians recognized the psychological benefit of companion animal interaction for human healing and in recent decades the 'human - companion animal bond' has been widely explored. This touches not only on 'pets as therapy' in hospitals or care homes but the widespread psychological and social impact of pets in society [33].

## Competing interests

The author is a member of the Bayer CVBD World Forum. The WSAVA One Health Committee is funded through the WSAVA Foundation by a consortium of industry sponsors (Bayer Animal Health, Dechra, Hills Pet Nutrition, Intervet-Schering Plough Animal Health, Merial, Nestlé Purina, Novartis Animal Health, Pfizer Animal Health and Waltham).
